# A National Survey of Companion Animal Owners’ Self-Reported Methods of Coping Following Euthanasia

**DOI:** 10.3390/vetsci7030089

**Published:** 2020-07-07

**Authors:** Rachel Park, Kenneth Royal

**Affiliations:** Department of Clinical Sciences, North Carolina State University, Raleigh, NC 27606, USA; rmpark@ncsu.edu

**Keywords:** pet bereavement, euthanasia, coping, survey, pet loss, grief, human–animal interactions, companion animal

## Abstract

(1) Background: The human–animal bond is often regarded as a special relationship in which owners benefit from unconditional love and perceived understanding from their companion animal. Thus, end-of-life decisions such as euthanasia may inflict significant emotional impact upon the companion animal owner and result in a complicated grief response. The purpose of this study was to examine the methods American companion animal owners utilize to cope with loss following companion animal euthanasia. (2) Methods: A total of 340 companion animal owners with experience euthanizing a companion animal completed an online survey asking how they found comfort after the loss of their companion animal. (3) Results: A total of 74.7% noted that they mourned privately, 58.2% sought social support, 32.1% adopted a new companion animal, 12.4% relied on faith or prayer and 0.9% participated in a support group. (4) Conclusions: Grief associated with companion animal loss is important. As a majority of clients that euthanized their companion animal mourn privately, the veterinary community must work towards identifying and providing appropriate, accessible social resources for bereaved companion animal owners to utilize, if desired.

## 1. Introduction

In Western culture, companion animals are often considered integral members of the family. Through this in-group membership status, companion animals may facilitate social support [1] and enhance physical and mental well-being for their owners [2,3]. The human–animal bond is often regarded as a special relationship, where owners benefit from unconditional love and perceived understanding from their companion animal. As a result, owners and companion animals may spend a large portion of their time together (e.g., traveling, working, exercising, and playing together) and share in major life milestones (e.g., holidays and celebrations).

Unfortunately, as our four-legged companions have relatively short lifespans, an inevitable aspect of companion animal ownership is companion animal loss and millions of Americans will mourn for their companion animals annually [4]. Grieving a companion animal can be an intense emotional process, resulting in feelings of sadness, despair, and numbness. The grief experienced by bereaved companion animal owners may mirror that of a human loss [5], particularly for companion animal owners with strong attachment to their companion animal [6,7].

However, in contrast to human loss, companion animal loss typically lacks socially endorsed rituals or memorials (e.g., calling hours, funerals, bereavement leave, social outreach cards, and covered dishes) and the absence of these practices may suggest that companion animal loss is a lesser reason to grieve [8,9]. Bereaved companion animal owners may face additional social challenges, as individuals within their support system may not understand the significance of their loss [10,11,12]. These experiences further invalidate the grief response, as bereaved companion animal owners fear judgement from others and that their feelings are not openly acknowledged or valued (i.e., disenfranchised grief) [13,14].

Concomitantly, companion animal owners are often responsible for making the decision to euthanize. Euthanasia may be decided upon when companion animals are suffering from untreatable or chronic pain (e.g., injury, illness, and aging). In companion animal medicine, the preferred method of euthanasia is performed through barbiturate overdose [15] in order to offer a quick and painless death to the animal. Veterinarians can provide medical advice, but the euthanasia decision must be made by the owner. However, even when owners understand that euthanasia is the best option for their companion animal, they often report that their close bond with the companion animal made their decision difficult [16].

As the decision to end a companion animal’s life may emotionally impact the companion animal owner, the experience of euthanasia can result in a complicated grief response. Previous research has indicated that nearly one-third of companion animal owners that euthanized their companion animal reported experiencing profound or severe grief [17,18]. This subset of bereaved companion animal owners may struggle with additional feelings of guilt and regret, particularly over the circumstances surrounding their decision (e.g., financial and timing) [8,19]. Thoughts that these companion animal owners may struggle with include whether the decision they made was right or wrong and whether it aligned with what their companion animal would have “wanted” [20]. Although emotional responses are more common, some bereaved companion animal owners may experience physical symptoms (e.g., vomiting, trembling, nightmares, dizziness, and black outs) [21]. Additionally, individuals experiencing severe or profound grief are at a heightened risk of suffering from psychological disorders (e.g., depression and anxiety) [22]. Therefore, companion animal owners that had to make the decision to euthanize may benefit from greater access to support that can assist in facilitation of coping mechanisms.

Prior studies have identified that bereaved companion animal owners may manage their grief utilizing various coping style. Companion animal owners may benefit from social support following their loss [4,10]. Social support can be provided directly from people present in the companion animal owner’s life or through alternative interventions (e.g., veterinarians, support groups, counseling, and pet loss support hotlines) [8,20,23]. In addition to social support, the use of continuing bonds (e.g., memorials/funeral services, reminiscing over memories, and writing letters to the deceased) may provide bereaved companion animal owners with comfort [6,24,25]. Other methods of coping include relying on religion, participating in community service and connecting with other animals [18,21,26].

The purpose of this study was to explore experiences of companion animal euthanasia and associated coping mechanisms utilized among a large and diverse sample of American companion animal owners. Although numerous previous studies have investigated companion animal bereavement, each were conducted at a single practice [27,28], within a limited geographic region [5,29,30], utilized convenience samples [31,32] or recruited directly from companion animal bereavement support groups or websites [10,25,26]. Further, these studies have traditionally lacked gender diversity, as they have been comprised of predominantly female participants. These factors have limited the ability to generalize the findings from previous research. The present study offers data from a national sample with approximately equal gender representation. It is our belief that the findings from a national sample of companion animal owners will provide more accurate insights about the coping mechanisms that companion animal owners typically employ after companion animal euthanasia.

## 2. Materials and Methods

Amazon Mechanical Turk (mTurk) is a crowdsourcing marketplace via Amazon.com in which participant ‘workers’ are compensated through nominal payments to complete surveys. Extant research has noted that mTurk samples provide socioeconomically and culturally diverse participants [32]. For this reason, mTurk was utilized in an effort to recruit a sample of companion animal owners with euthanasia experience reflective of the United States demographic population. Furthermore, mTurk data quality is comparable to other platforms for obtaining survey participants [32,33].

For the present study, a sample of 800 mTurk participants who identified as companion animal owners were obtained in order to yield a margin of error within 3.5%. All participants were at least 18 years old and were legal residents of the United States. Each participant received a nominal fee ($0.50) for participating in the survey.

The questionnaire asked companion animal owners a qualifying item “Have you ever had to euthanize a pet?”. This item was intended to estimate the percentage of American companion animal owners who have experienced companion animal euthanasia. Those that responded ‘yes’ were then asked “How did you find comfort after your loss?” and asked to select each strategy from a pre-determined list informed by the research literature [21,26,34]. Those that answered ‘no’ were excluded from the analysis. A series of demographic items using U.S. Census categories were also included for those that answered ‘yes’ to the aforementioned item. Ethical approval to conduct the study was granted by the university’s Institutional Review Board (IRB) (Protocol #5626).

Descriptive statistics (e.g., counts and percents) were calculated for all items. Results were compared by key demographic criteria, including gender, race/ethnicity, age group and geographic region. Comparisons were performed using chi-squared tests. All analyses were performed using SPSS (version 24) statistical software (Armonk, NY, USA).

## 3. Results

Of the 800 participants initially invited to participate in the study, 340 (42.5%) reported to have previously euthanized a companion animal. Table 1 provides a breakdown of the demographic characteristics of the sample frame.

Respondents who indicated that they had euthanized a companion animal were asked “how did you find comfort after your loss?” and instructed to select all that apply. Respondents’ answers are presented in Table 2.

A total of 22 respondents noted “Other” and were asked to specify. Responses consisted of the following themes: found no comfort (*n =* 3); alcohol (*n =* 3); acceptance of death (*n =* 3); distracting self (*n =* 2); did not seek comfort (*n =* 2); increased interactions with other family pets (*n =* 2); volunteered at an animal shelter (*n =* 1); memorialized pet (*n =* 1); cathartic crying (*n =* 1); reflecting on joy animal brought the household (*n =* 1); thought of how the animal was not suffering anymore (*n =* 1); read a book about coping with pet loss (*n =* 1); and did not need comfort (*n =* 1). With respect to response differences based on demographic criteria, no statistically significant differences were discernible.

## 4. Discussion

The objective of the present study was to explore the experiences of companion animal euthanasia and associated coping mechanisms utilized among a large and diverse sample of American companion animal owners. Of the 800 companion animal owners surveyed, 340 (42.5%) reported experience with companion animal euthanasia. Although the present study design has limitations (e.g., lack of racial diversity and potential selection bias), it would be fair to assume that a large majority of Americans have experience with companion animal euthanasia, given that nearly 68 million American households contain companion animals [35,36]. Therefore, understanding the experience of companion animal loss from euthanasia is important.

With respect to coping with companion animal loss after euthanasia, most participants (74.7%) noted that they mourned privately. Previous research has demonstrated that bereaved companion animal owners frequently practice emotional distancing and social isolation [27,37]. Bereaved companion animal owners struggling with their emotions may tend to avoid others while processing their experience [20]. Self-isolation may be a choice made by bereaved companion animal owners, but another possibility is that this behavior is a result of the disenfranchised experience of companion animal loss. This finding is alarming, as there are negative health and social consequences associated with companion animal owners that experience disenfranchised grief. Social constraints can be harmful to bereaved companion animal owners, who may become less capable of coping when they cannot outwardly express their emotions with others. Internalized grief by bereaved pet owners has been associated with depression, poor physical health and increased stress [10,38]. In a qualitative study, Tzivian and Friger [21] conducted interviews with companion animal owners who recently euthanized their companion animal. In these accounts, companion animal owners expressed fear in sharing their grief with loved ones, as they did not feel they could understand their experience. Therefore, feelings of fear, loneliness and social rejection may foster an environment of isolation resulting in bereaved companion animal owners becoming incapable of asking for social support [20].

Another sizeable contingent of participants (58.2%) reported seeking support from family and friends. The majority of bereaved individuals (i.e., those suffering a human death) rely on social support from friends and family to cope with their grief and this support is a strong predictor of positive psychosocial outcomes following loss [39,40]. As the human–animal bond may be comparable in strength to human relationships (e.g., familial, friendship) [41], the benefits from coping strategies used following human loss may be similar for bereaved companion animal owners. Bereaved companion animal owners that made the decision to euthanize may particularly benefit from post-traumatic growth (i.e., positive emotions or behaviors following a traumatic experience). Within the nuclear family, relationships may strengthen from enduring the experience together. Family members report feeling a sense of unity and appreciation for one another following the loss of a companion animal [42]. To summarize, a large proportion of respondents utilized social support from family and friends following their companion animal loss and this may be considered a healthy coping mechanism.

Interestingly, nearly one-third of participants (32.1%) noted that they adopted a new companion animal. This figure aligns with the larger population of bereaved companion animal owners (28%) [18] but contrasts with a previous qualitative study investigating coping strategies following canine euthanasia (14%) [21]. The difference in sampling methodology may contribute to the disparity observed. Tzivian and Friger [21] interviewed Israeli dog owners in the 2–3 weeks following euthanasia. In addition to the difference in population characteristics investigated (i.e., country of residence, companion animal type), the present study did not identify the time frame that had elapsed since the experience with euthanasia. Time frame may be an important consideration for this item, as the suggestion of adopting another animal shortly after loss may be viewed as inappropriate by some companion animal owners [19]. Companion animal owners that adopt another companion animal following their loss do not view the new companion animal as a replacement to the previous relationship but identify the new companion animal as a helpful coping mechanism [18]. Companionship and the act of caring for another companion animal may offer psychological comfort to bereaved companion animal owners [20].

A small percentage of participants (12.4%) noted that they prayed and/or relied on their faith to seek comfort. Compared to previous studies, this percentage was significantly lower. Lee [26] found that nearly 72.1% of bereaved companion animal owners engaged in positive religious coping with 83.1% praying for their companion animal’s well-being in the afterlife. However, in a separate study investigating the role of religion in the companion animal grief process, only 36.8% of participants reported praying for their companion animal [18]. The lower rate in our study may be attributed to the demographic characteristics of participants, particularly age, as younger adults are more likely to experience death anxiety but less likely to engage in religious coping during times of grief [43]. Companion animal owners that do endorse faith benefit from increased resilience and comfort during the grieving process [20,44].

Surprisingly, very few participants (0.9%) noted participating in a support group (in person or virtual). Support groups may offer an environment for bereaved companion animal owners to share their grief with others that can relate to the loss of this relationship. In addition to expanding the bereaved companion animal owner’s social network, those individuals that participate in support groups may benefit from decreased negative emotions (e.g., guilt and depression) and increased psychosocial outcomes [6,34]. For these reasons, support groups are a common recommendation from veterinarians following the death of a pet [23,34,45]. Despite the benefits to participants, the present results suggest that this is not a widely utilized intervention by companion animal owners. Possible explanations for this finding include that support groups may not be available in companion animal owners’ geographic area, or support groups are available but bereaved companion animal owners may not have knowledge of their existence.

### 4.1. Contributions to the Literature

The present study contributes to the literature in two primary ways. First, this study offers findings that are built upon a strong methodological approach. That is, unlike prior studies that investigated companion animal bereavement, this study utilized a diverse (e.g., age, gender, geographical regions) and national sample of participants, thus increasing the likelihood that the findings may be generalizable. Second, this study offers substantive findings that conflict with previous literature. It was discerned that 43% of companion animal owners surveyed had experience with companion animal euthanasia, a figure that is the first to estimate a national rate for pet euthanasia. Further, this study investigated a subset of bereaved companion animal owners that experienced euthanasia with their companion animal and identified the primary ways in which individuals cope with companion animal loss after euthanasia. We also argue that because of the sample frame selected, the findings of this study are more likely to represent true estimates on companion animal bereavement coping styles.

### 4.2. Limitations

In the present study, an online survey was utilized to collect data on coping styles used by companion animal owners following a euthanasia experience. A crowd-sourcing platform (Amazon mTurk) was chosen to recruit a diverse national sample. However, this methodology may include potential selection bias, as participants made the decision to complete the survey. Additionally, participant workers must have access to the internet and a computer to complete the questionnaire.

The survey did not collect information on companion animal demographics (e.g., species, age, and length of ownership). It is unknown which specific animals respondents reported to have previously euthanized, as the survey simply referred to “pets”. We assume that most companion animals were dogs and cats but cannot speak to specifics. Similarly, euthanasia-related demographics (e.g., reasoning, time elapsed since euthanasia procedure, and who was responsible for the euthanasia decision) were not addressed in the survey. Without this information, it is not possible to determine whether coping styles utilized are attributed to grief resulting from the euthanasia decision or the companion animal loss itself. It is also unknown the extent to which the demographic characteristics of the sample are representative of the American populace, as true population parameters for companion animal owners in the United States are not known. This limitation is persistent in virtually every study involving companion animal owners/ownership. Finally, although the sample obtained was generally diverse with respect to key demographic variables, the race/ethnicity variable was less diverse in this study, as the majority (92.6%) of respondents were White. The extent to which companion animal ownership experiences may differ based on race/ethnicity poses a potential limitation to this work.

### 4.3. Future Directions

In the present study, the majority of participants indicated that they mourned their loss privately. This coping mechanism may be harmful to companion animal owner well-being and foster a cycle of isolation during a time when social support is needed. While social support groups for bereaved companion animal owners exist, participants did not widely utilize this coping mechanism. There is an additional need for research that explores the reasoning behind why companion animal owners choose to engage (and not engage) in certain coping practices. Future studies should further evaluate effective coping strategies for bereaved companion animal owners. Furthermore, there is a gap in knowledge surrounding anticipatory grief in companion animal loss due to euthanasia and this should be investigated.

## 5. Conclusions

The purpose of this study was to examine the coping methods companion animal owners utilize after experiencing companion animal euthanasia. Results of the present national survey indicate that 43% of companion animal owners have experience with companion animal euthanasia. Companion animal owners largely mourn privately and/or with family and friends. A small contingent of companion animal owners rely on faith/prayer as coping mechanisms, and very few (<1%) join emotional support groups, a best practice for coping with pet loss. Clients that euthanize their companion animal may benefit from additional social support (e.g., support groups, counseling, and pet loss hotlines) [23,46] or educational materials (e.g., grief literature) [46]. Results from this study may be used as a basis to incorporate these support tools into standard practice for euthanasia clients.

## Figures and Tables

**Table 1:** Demographics Characteristics of Sample.

Variable	*N*	%
Gender		
Male	193	56.8
Female	147	43.2
Race/Ethnicity		
Asian or Pacific Islander	8	2.4
Black or African American	9	2.6
Hispanic or Latino	5	1.5
Other	3	0.9
White	315	92.6
Age (year)		
≤20	9	2.6
21–30	139	40.9
31–40	98	28.8
41–50	53	15.6
51–60	31	9.1
≥61	10	2.9
Geographic Region		
Midwest	88	25.9
Northeast	59	17.4
South	130	38.2
West	63	18.5

**Table 2:** Actions taken to find comfort after pet euthanasia.

Coping Mechanism Utilized	*N*	%
Mourned privately	254	74.7
Sought support from family/friends	198	58.2
Adopted a new pet	109	32.1
Prayed/relied on faith	42	12.4
Other	22	6.5
Participated in a support group	3	0.9
